# The short-term efficacy of adventitial inversion with graft eversion anastomosis for the reconstruction of the aortic sinus in the root treatment of aortic dissection

**DOI:** 10.3389/fcvm.2022.845040

**Published:** 2022-08-22

**Authors:** Feng Gao, Zepeng Shi, Xuezhi He, Yang Gao, Xijing Zhuang, Lei Shi, Wenjun Wang, Wei Liu

**Affiliations:** Department of Cardiovascular Surgery, Dalian Municipal Central Hospital, Dalian, China

**Keywords:** anastomosis technique, aortic dissection, aortic root, efficacy, short-term

## Abstract

**Background:**

The surgical approaches for a mildly affected aortic sinus (AS) are varied and controversial. Here, the AS was reconstructed using the extended adventitial inversion with graft eversion anastomosis technique before its perioperative and short-term efficacy was compared with that of the vascular grafts that wrap the aortic wall and the right atrial shunt technique, providing a new basis for surgical management strategies.

**Method:**

A total of 101 patients with mildly affected AS were enrolled in the clinical trial. The extended adventitial inversion suture and the graft eversion anastomosis technique was performed in group A. Aorta wrapping and the right atrial shunt technique were performed in group B. The primary endpoints were reoperation-related events and fatal events related to the aorta, while the secondary endpoints were the duration of surgery and structural changes in the aortic root. Cardiac ultrasound and aortic computed tomography angiography examinations were performed before surgery, 2 weeks after surgery, and 1 year after surgery.

**Results:**

Compared to group B (*n* = 56), group A (*n* = 36) had a significantly shorter duration of surgery (the time from skin incision to skin closure) and a reduced time from shutdown to skin closure (*P* < 0.05). Cardiovascular ultrasound examinations performed at follow-up 12 months after surgery and 2 weeks after surgery revealed a significant increase in the diameter of the aortic sinotubular junction (STJ) of group B (*n* = 50) (*P* < 0.05). The extended adventitial inversion suture and the graft eversion anastomosis technique (*n* = 33) performed better than Aorta wrapping and the right atrial shunt technique in terms of persistence of the false lumen closure effect, anastomotic leakage, and reduction in aortic valve (*P* < 0.05), and there was a significant difference between the two groups in terms of the incidence of events related to reoperation (*P* < 0.05).

**Conclusion:**

Compared with the aorta wrapping and the right atrial shunt technique, the extended adventitial inversion suture and the graft eversion anastomosis technique allow shortening of the operation time and preventing near-term dilation of the STJ, with improved safety and an improved short-term surgical effect.

## Introduction

Eliminating a false lumen by replacing the aorta in the area of the injury with vascular grafts is the main treatment method for acute A-type aortic dissection (AAAD). However, it is extremely difficult to achieve rapid, accurate, and effective suturing and hemostasis due to intraoperative vascular inflammatory edema and increased tissue brittleness (1). In addition, completing an effective hemostasis procedure by adding needles after exposure is proven to be problematic due to the dramatic change in blood flow at the aortic root and the deep anatomical level, even if the bleeding site is clear. As such, investigating how to effectively reduce the risk of anastomotic bleeding at the root and increase overall surgical safety has become a research focus for many clinical treatment strategies.

The root of aortic dissection in the case of an affected aortic sinus (AS) is generally treated using the technique that involves vascular grafts of the aortic wall and a right atrial shunt to reduce the risk of subsequent bleeding. However, the condition is often discovered late, making it difficult to treat, since the anastomotic leakage becomes completely covered and buried (2). The selection of the appropriate surgical strategy is more complex for patients with mildly affected AS, and the available strategies have become a focus of clinical scholars seeking to determine how to reduce the risks related to surgery and how to deliver maximum long-term benefits while maintaining normal aortic root function as much as possible. In recent years, the method that involves “bare” treatment of the aortic root has emerged, and the advantages of convenient hemostasis using needles after the completion of the anastomosis have encouraged further exploration of anastomosis-based treatment.

Our center further improved the original graft eversion anastomosis technique by expanding the suturing depth of the adventitial inversion and integrating it with the concepts of eversion and anastomosis. The aim of the present study is to summarize the characteristics and advantages of this technique and to explore whether there is a difference between this technique and the aorta wrapping and the right atrial shunt technique in terms of the short-term effect on root treatment. The paper is expected to provide a reference for the selection of the appropriate surgical treatment strategy (3).

## Patients and methods

### Study design and study population

This was a single-center retrospective study involving 1: 1.5 randomly assigned intent-to-treat groups in the Cardiac Great Vascular Surgery Center at Dalian Central Hospital, Liaoning Province, China, a tertiary academic teaching hospital. A total of 101 patients were enrolled in this study, with nine patients excluded after the randomization process because they refused a postoperative reexamination and requested to be discharged and because valve replacement and concurrent coronary artery bypass grafting were required during intraoperative examination of any damage to the aortic valve structure. This left a total of 92 patients, 36 assigned to group A and 56 to group B. All eligible patients were enrolled from March 15, 2017, to October 30, 2020. The postoperative follow-up rate of the patients was 100% and the last follow-up date was November 15, 2021.

The subjects of this study were mainly patients with AAAD with mildly affected AS who met the following inclusion criteria: (1) patients with dissection that affected the AS or a fissure in the AS and with an AS diameter <45 mm, (2) patients with moderate or mild regurgitation of the aortic valve caused by the involvement of the junction of the aortic valve, (3) patients with good structural integrity of the aortic valve, and (4) patients with tight and intact adventitia at the aortic root. Meanwhile, the exclusion criteria were as follows: (i) patients who underwent other cardiac surgery at the same time (including coronary artery bypass grafting and combined valve replacement), (ii) patients without ultrasound or computed tomography angiography (CTA) results at the preoperative and/or postoperative follow-up stages, and (iii) patients without postoperative follow-up data. All patients received detailed information on the surgical method and were asked to sign the informed consent form for inclusion in the study before the procedure, with the patients enrolled in the study the day after the completion of the surgery (after admission) and grouped according to the mode of management of the aortic root. All procedures were performed by the same senior surgeon, with a total of five cardiac surgeons who participated in the operations. Ethical approval was obtained from the center’s medical ethics committee and institutional review board, as well as written consent, according to the Declaration of Helsinki.

### Routine operation procedures

Cardiopulmonary bypass was established through the conventional axillary artery, femoral artery, and right atrium two-step tubes. Left cardiac drainage was placed in the right upper pulmonary vein, with arterial femoral and venous cannulation used in the case of unstable circulation. During the procedure, a pericardium drainage tube and a mediastinal drainage tube were housed and a disposable thoracic drainage bottle was connected to record the drainage volume. A postoperative routine admission to the intensive care unit was arranged for follow-up intensive care treatment.

### The extended adventitial inversion suture and graft eversion anastomosis technique

**FIGURE 1:**
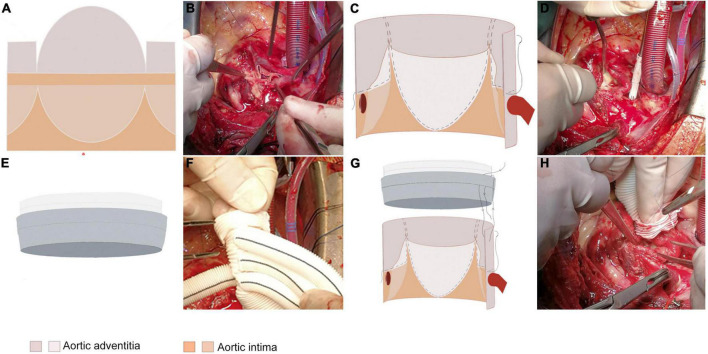
The extended adventitial inversion suture and graft eversion anastomosis technique. **(A,B)** Only the aortic intima is transected 0.5 cm above the STJ while leaving the aortic adventitia intact. The reserved aortic adventitia is cut along the three valve junctions longitudinally along the aortic axis to the edge of the transected aortic intima. The shape of the aortic adventitia is trimmed according to the shape of the three aortic sinuses such that it inverses toward the base of the sinus with the non-coronary sinus reaching the base of the sinus and the left/right coronary sinuses reaching the upper edge of the coronary ostia. **(C,D)** The trimmed aortic adventitia is inversed toward the three coronary sinuses and secured with 1–2 continuous sutures for fixation. **(E,F)** Eversion of the proximal end of the graft outwards by 1 cm. **(G,H)** Performing the vascular grafts eversion, insertion into the aortic lumen to align the edges of the two tubes, and suturing the vascular grafts to the aortic wall 1–2 times with an edge distance of 0.5 cm.

The extended adventitial inversion suture and graft eversion anastomosis technique was performed in group A as follows. In the cooling phase of cardiopulmonary bypass, the aortic root was treated by annular trimming of the aortic intima to the plane above the sinotubular junction (STJ) by cutting along the longitudinal axis of the valve junction and trimming the adventitia of the aortic (Figures 1A,B). Continuous mattress suturing was performed over the non-coronary sinus and the left and right coronary ostia using sutures with spacers, and the structure of the aortic root was completely exposed by traction. The adventitia of the aorta was inverted so that it could be attached to the intima of each coronary sinus. Continuous suturing was performed at the junction of the right sinus and the non-coronary sinus, the latter sutured to the level of the aortic annulus and the traction pad position held above the coronary ostia for the left and right coronary sinuses (Figures 1C,D). The junction of the avulsed aortic valve was fixed by adventitia reduction, with the closure tested by injection of water. Vascular grafts of an appropriate diameter were selected, proximal ends of which were inverted and inserted into the aortic root for continuous suturing (Figures 1E–H). Finally, the anastomotic stoma was checked by perfusion, with mats applied to reinforce the local bleeding points (3).

### Aorta wrapping and right atrial shunt technique

The aorta wrapping and right atrial shunt technique was performed in group B as follows. The anterior wall of the aorta was dissected longitudinally and semitransected close to the STJ to preserve the longitudinal continuity of the posterior wall of the aorta. Treatment of the aortic root was performed during the cooling period, with the junction of the avulsed aortic valve reduced and fixed through junction suspension, followed by a closure test using water injection. Then, end-to-end anastomosis was performed for vascular grafts with STJ and the distal end of the aortic circumferential integrity area. Following cardiac re-beating and local hemostasis, the aortic wall was trimmed and retained, and continuous suturing was performed toward the proximal end to construct the vascular grafts wrapped in a sac. A gap of approximately 2 cm was reserved when the cystic cavity was sutured on the right atrium side, with the right atrium fixed using auricular forceps to complete the right atrial shunt and anastomosis of the aortic sac once the circulation was stable. Pericardial patches could be applied to enlarge the sac wall to complete the setting of the shunt and anastomosis in the case where the tension of the sac wall was too high. It was essential to check for the presence of anastomotic stenosis, uneven tension, and too high right atrial tension following completion of the anastomosis (4).

### Study design and definition of variables

The primary endpoints included reoperation-related events and fatal events related to the aortic, with the former including a worsening of aortic valve regurgitation, anastomotic leakage of the aortic root, formation of vegetations or mediastinal infections of the aortic root, new pericardial effusion of medium volume or greater, and false lumen recurrence of the aortic dissection. The first valid event was recorded in terms of the occurrence time. Meanwhile, the secondary endpoints included the duration of the surgery and any structural changes to the aortic root.

The variables to be recorded included the following: (1) general preoperative information, including sex, age, hypertension, diabetes, smoking history, drinking history, surgical method, valve function, AS involvement and valve junction, and coronary ostia involvement; (2) surgical aspects, including myocardial infarct time, circulatory arrest time, total duration of operation (from skin incision to skin closure), the time from the skin incision to shut down and the time from shutdown to skin closure; (3) auxiliary examination aspects, including the diameter of the AS, the aortic STJ, the aortic annulus, the left ventricular end diastolic, the right atrial end systolic, and the left ventricular ejection fraction, which were evaluated using ultrasound examinations before surgery, at two weeks after surgery and at other. Cardiac ultrasound and contrast-enhanced CTA were performed 12 months after surgery, with the time of occurrence of any fatal event related to the aortic among the patients recorded.

The patients were randomly assigned in a 1:1.5 ratio using the random number method. Following the completion of the sampling by an independent researcher, the surgeons were informed of the scope of the lesions after the patients had received the relevant examinations on admission, with the related information recorded once the patients and their families had signed the informed consent form. Given the nature of the intervention, patients, surgeons and researchers were all aware of the treatment methods.

### Statistical analysis

Statistical analysis was performed using SPSS 25.0 software, with all continuous variables subjected to the Kolmogorov–Smirnov normality test. Measurement data that followed normal distribution were expressed as mean ± standard deviation (*X* ± s), with two independent sample *t*-tests used for intergroup comparisons, while data not following normal distribution were expressed in terms of median (interquartile range), with the Wilcoxon rank sum test used for intragroup comparisons and a Mann–Whitney rank test for intergroup comparisons. Meanwhile, a chi-square (χ^2^) test was used for the inter-group comparison of the enumeration data, with the Kaplan–Meier method used to draw survival curves for statistical analysis of any fatal events related to aortic and the first events related to reoperation during follow-up. A logarithmic rank test and a Breslow test were used to compare the differences between the two groups of patients, with a *P* value < 0.05 considered statistically significant.

## Results

### Baseline characteristics

The baseline demographic and structural characteristics of the aortic root of the patients are shown in Table 1. There were no significant statistical differences between the two groups of patients prior to surgery.

**TABLE 1:** Baseline characteristics.

Variable	Group A (*n* = 36)	Group B (*n* = 56)	*P* value
Sex(male)	21(58.3)	34(60.7)	0.820
Age, years	56.58 ± 13.72	57.50 ± 11.58	0.731
Hypertension	28(77.8)	46(82.1)	0.606
Diabetes	6(16.7)	12(21.4)	0.574
Smoking	10(27.8)	16(28.6)	0.934
Drinking	9(25)	12(21.4)	0.690
COPD	1(2.8)	3(5.4)	1.000
CAD	3(8.3)	5(8.9)	1.000
Marfan syndrome	0(0)	2(3.6)	0.518
Hematemesis/hematochezia	0(0)	0(0)	1.000
Acroparesthesia/extremity dysfunction	8(22.2)	10(17.9)	0.606
Limb ischemia	6(16.7)	9(16.1)	1.000
History of syncope	2(5.6)	6(10.7)	0.475
History of cardiac surgery	0(0)	0(0)	1.000
*Scope of surgery*			0.174
Ascending aorta replacement	16(44.4)	26(46.4)	
Ascending aorta replacement and half aortic arch replacement/reconstruction	13(36.1)	17(30.4)	
Ascending aorta replacement, aortic arch replacement and stenting	17(47.2)	13(23.2)	
Aortic regurgitation (mild/moderate)	16/20	33/23	0.174
*Aortic sinus involvement*			
Non-coronary sinus	30(83.3)	44(78.6)	0.639
Right coronary sinus	21(58.3)	25(44.6)	
Left coronary sinus	6(16.7)	5(8.9)	
*Aortic valve junction involvement*			
Right-non-coronary valve junction	19(52.8)	40(71.4)	0.786
Left-non-coronary valve junction	3(8.3)	4(7.1)	
Right-non-coronary valve and left-non-coronary valve junction	3(8.3)	8(14.3)	
*Coronary artery ostia involvement*			
Neri A	6(16.7)	12(21.4)	0.533
Neri B	7(19.4)	9(16.1)	
*Cardiac echocardiography*			
AS (mm)	37[34–39.75]	36[32.25–39.75]	0.327
STJ (mm)	38[34–40]	38[34–40]	0.932
Aortic annulus (mm)	25[23–26]	26[23–26.75]	0.232
LVD (mm)	50[47–51]	49[46.25–51.75]	0.478
RAD (mm)	24[24–38]	26.5[25–30]	0.304
LVEF (%)	46[44–55.75]	48[44.25–52]	0.590
*Time associated with surgery*			
Myocardial block time	83[66–99]	80.5[71–96.75]	0.773
Time from skin incision to shutdown	278.95[254.1–316.8]	306.1[279.2–332.35]	0.052

Values are expressed as n(%), mean ± SD, or median[interquartile range]; Neri classification (5): A: Normal coronary artery opening; B: Coronary opening hematoma. COPD, chronic obstructive pulmonary disease; CAD, coronary artery disease; AS, aortic sinus; STJ, sinotubular junction; LVD, left ventricular diameter; RAD, right artial diameter; LVEF, left ventricular ejection fraction.

### Operation time

**FIGURE 2:**
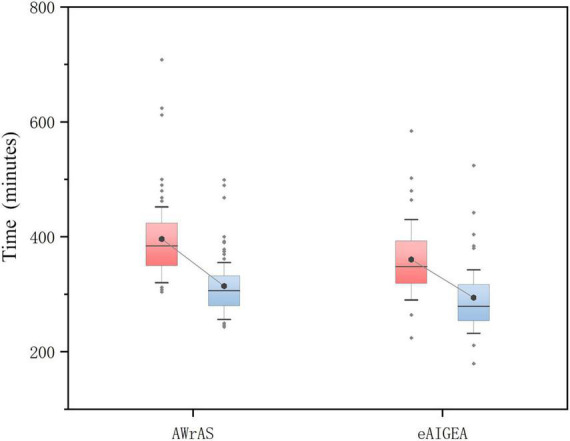
The total duration of the surgery and the time from shutdown to skin closure. The total duration of the operation in the group A was significantly shorter than in the group B (*P* = 0.022), while there was a significant difference in the time from shutdown to skin closure between the two patient groups (*P* < 0.001; box diagram).

Compared to group B, there were significant statistical differences in the total duration of surgery (from skin incision to closure) and the time from shutdown to skin closure (*P* < 0.05) in group A (Figure 2), while there were no significant statistical differences in the remaining operations-related times (Table 1).

### Follow-up

**FIGURE 3:**
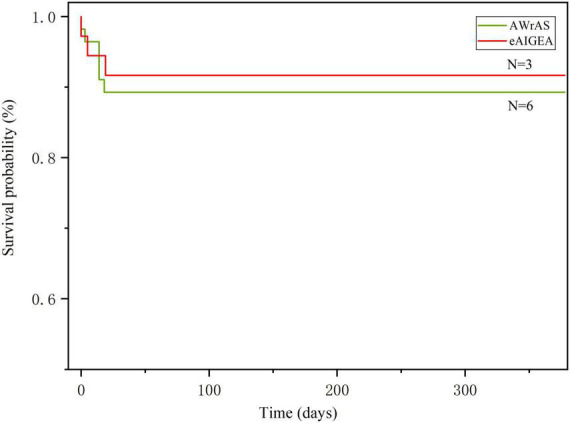
The Kaplan–Meier method was used for plotting the survival curve. The log-rank (*P* = 0.69) and Breslow (*P* = 0.66) test results indicated that there were no differences in the short-term mortality rate between the two surgical methods.

The general follow-up time was 303–378 days, with an average of 339.9 ± 18.62 days. During the follow-up period, nine patients died from circulatory failure, all during postoperative hospitalization, which included three patients in group A and six in group B (two patients underwent secondary thoracotomy for hemostasis), with aortic-related mortality rate within 30 days after surgery 9.78%. One patient in group B died in a traffic accident during the follow-up period and, as such, the results of their last follow-up visit were included in the study. The results of the logarithmic rank survival curve log-rank test results (χ^2^ = 0.16, *P* = 0.69) and the Breslow test results (χ^2^ = 0.18, *P* = 0.66) indicated that there were no significant statistical differences between the two surgical methods in terms of the short-term postoperative mortality rate (Figure 3).

### Changes in cardiac structure during follow-up

The STJ of group B was significantly higher compared to that of group A two weeks after surgery (*P* = 0.002), and the STJ of group A did not show significant changes. Furthermore, no significant changes were observed in the other data on aortic roots related to both groups (Table 2).

**TABLE 2:** Changes in cardiac structure after surgery and at follow-up nodes.

	Ultrasonic structure index	Within 2 weeks after surgery	Follow-up nodes	*P* value
Group A (*n* = 33)	AS (mm)	35[33–40]	36[32–38.5]	0.307
	Aortic annulus (mm)	25[23–25.5]	24[23–25]	0.847
	STJ (mm)	39[35–40.5]	35[33–40]	0.083
	LVD (mm)	48[46–50]	48[46–49.5]	0.150
	RAD (mm)	24[22–40]	24[22–41]	0.537
	LVEF (%)	45[44–52]	46[44–52]	0.858
Group B (*n* = 50)	AS (mm)	38[34–41.25]	37[34–40.25]	0.317
	Aortic annulus (mm)	25[23–26]	25[23–26]	0.480
	STJ (mm)	42[36–48]	44[38–49.25]	0.002
	LVD (mm)	48[45.75–50]	48[45–50]	0.344
	RAD (mm)	28[26–36]	28.5[26–36]	0.439
	LVEF (%)	48[44.75–50.25]	48[45–50]	0.705

AS, aortic sinus; STJ, sinotubular junction; LVD, left ventricular diameter; RAD, right artial diameter; LVEF, left ventricular ejection fraction.

**FIGURE 4:**
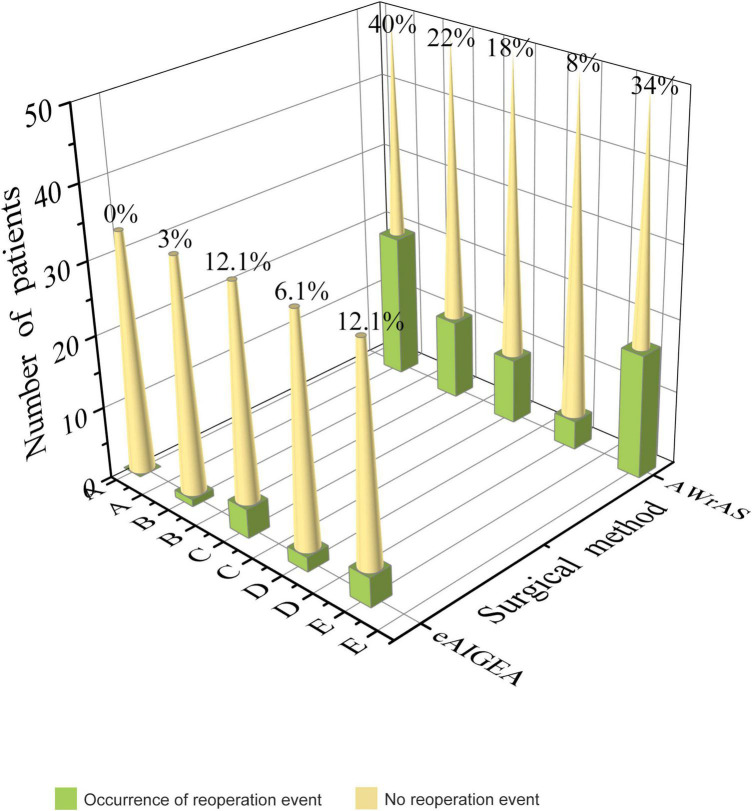
Reoperation-related events: A) recurrence of the false lumen in the aortic root dissection (*P* < 0.001), B) anastomotic leakage of the aortic root (*P* = 0.016), C) pericardial effusion of moderate volume or above (*P* = 0.471), D) a mediastinal infection or infective endocarditis (*P* = 0.738), E) progression of aortic valve regurgitation during the follow-up compared with that after surgery (*P* = 0.025).

No aortic valve regurgitation was observed among the patients during the intraoperative instant water injection test, cardiac ultrasound and CTA examinations were routinely performed within two weeks after surgery and at other follow-up points. Reoperation-related events of the patients in the two groups are shown in Figure 4. Compared to the results of a postoperative reexamination, four patients in group A exhibited varying degrees of progressive aortic valve regurgitation, while a total of 17 patients in group B exhibited a significant progression of valve regurgitation, with a significant statistical difference in the maintenance of the valve repair effect between the two groups (*P* = 0.025). Closure of the false lumen in dissection and prevention of anastomotic leakage were significantly better in group A than in group B (*P* < 0.05) (Table 3 and Figure 4).

**TABLE 3:** Reoperation-related events.

Evaluation items at follow-up nodes	Group A (*n* = 33)	Group B (*n* = 50)	*P*
A: Recurrence of false lumen of aortic root dissection	0	20	<0.001
B:Stomal leak of aortic root	1	11	0.016
C. Pericardial effusion of moderate volume or above	4	9	0.471
D: Mediastinal infection or infective endocarditis	2	4	0.738
Aortic valve regurgitation:			
Presence of aortic regurgitation 2 weeks after surgery	5	12	0.328
E: Progression of aortic valve regurgitation at follow-up nodes compared with that after surgery	4	17	0.025

**FIGURE 5:**
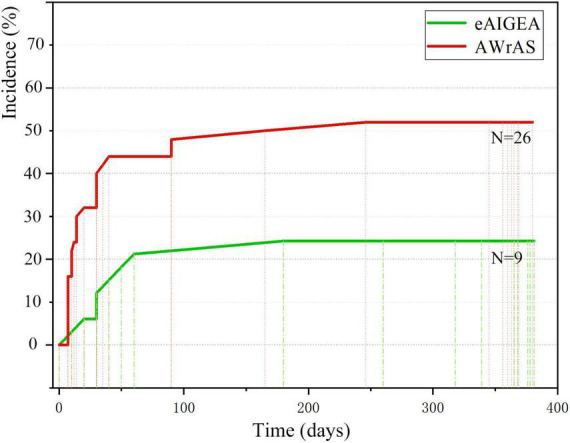
The incidence time curve of the first reoperation events.

The first occurrence time of reoperation-related events between the two groups was recorded (primarily the time of the first occurrence for patients with multiple reoperation-related events), with the incident time curve of the first reoperation-related event plotted accordingly (Figure 5). The log-rank test (χ^2^ = 7.08, *P* < 0.01) and Breslow test (χ^2^ = 8.12, *P* < 0.01) results indicated that there were significant differences in the incidence of events related to early postoperative reoperation between the two groups.

## Discussion

In terms of brittle and easy-to-crack intima and the progressive decline in platelets of acute AAAD edema with AS involvement, to reduce the risk of anastomotic avulsion and needle bleeding and restore valve characteristics, anastomotic stoma wrapped in the aorta wall and a shunt into the right atrial through a sac are generally applied in conjunction with a valve junction suspension technique to improve the surgical safety (6–9). This approach appears not to conform to normal physiology, based on a perspective that considers only the anatomical structure, and vacuum suction of the right heart system will significantly increase the risk of right ventricular failure in patients when the shunt bypass cannot be closed by itself for a range of reasons. However, extremely high requirements are in place for surgeon specialized technical skill levels, team cooperation, and the development of platform hardware related to other relatively complex aortic root remodeling techniques. Due to this highly technical barrier, it is difficult to identify a standardized treatment method for the aortic root that not only balances surgical complexity, but also maximizes the benefits for the patients (10, 11).

The adventitial inversion technique potentially allows further strengthening of the intima and sealing and, as such, eliminating the false lumen. For patients with aortic regurgitation complicated by valve junction avulsion, homeopathic reduction and fixation of the aortic valve junction during adventitial inversion are a technically reliable and feasible method due to the tough and dense texture of the adventitia (12, 13).

In this research, to increase the safety and ensure the long-term postoperative efficacy of the procedure, suturing was further expanded to the dense connective tissue of the aortic annulus in the non-coronary sinus, with suturing to the normal tissue at the bottom of the dissection in the left and right coronary sinus was attempted. If the coronary artery ostia are affected by aortic dissection, adventitial inversion and suturing to the flap ring are required to close the coronary artery ostia and restore coronary blood supply by coronary artery bypass grafting for Neri C patients. This is also necessary in view of performing adventitial inversion and folding of the pad for continuous suturing following the reduction of the coronary artery ostia by full-thickness U-shaped transmural suspension with a needle and pad at the upper edge of the coronary ostia for patients with Neri A and Neri B, with the subsequent results found to be satisfactory in clinical application.

Intraoperatively, it is difficult to clearly reveal the anatomy and stop bleeding by adding needles to the rear wall after the completion of the anastomosis due to the deep position of the aortic root. Therefore, the “eversion anastomosis concept” must be applied, where elastic retraction of vascular graft eversion could tighten the anastomosis sutures and increase the area of contact with the aortic wall, thus making the fit closer and reducing the risk of bleeding from the gap. Furthermore, continuous eversion of vascular grafts after anastomosis could make bleeding points visible and positioning of oozing blood more accurate, thus facilitating hemostasis. Meanwhile, the underlining of the tension-free suture could effectively weaken the cutting effect of the sutures, making suturing more accurate and reliable (14, 15).

In terms of the suturing procedure, a large area of suturing was required in group B, which required a high technical level of suturing skill. During the operation, continuous suturing was required to artificially construct a large area of the sac wall, and additional right atrial anastomosis significantly prolonged the total operating time and increased the risk of surgery-related adverse events. As the sac wall wrapping and anastomosis operation were carried out primarily after the end of cardiopulmonary bypass, the time from end of the closure to skin closure was prolonged. However, there were no significant differences between the two groups of patients in terms of the time required for root treatment and simple anastomosis between the vascular grafts and the aorta, indicating that the extended adventitial inversion suture and graft eversion anastomosis technique is relatively simple and could significantly reduce the operating time.

In the process of aortic root molding, it is often necessary to balance the scope of the treatment and the postoperative effect and to select a treatment method that is more consistent with the anatomical structure and hemorrhological mechanics of the aortic root, thus minimizing the impact of the surgical procedure on the cardiac structure and functions, which is the current driving force behind the development of the aortic root molding technique. In this study, there were no significant differences between the two groups of patients in terms of cardiac structure on the ultrasound cardiogram. However, the STJ diameter in group B during follow-up was significantly increased compared to that within two weeks after surgery, with the difference in the STJ diameter during follow-up between the two groups of patients considered to be the result of the restrictive role of the vascular grafts, which were inserted using the eversion anastomosis technique in the aortic lumen, thus limiting the expansion of the STJ diameter.

Meanwhile, the impact of blood flow forced the vascular grafts to exhibit an axial elastic motion and passively tightened the sutures with the aortic root, which in turn further tightened the aortic root to counteract the deformation caused by the impact of blood flow. A portion of the blood kinetic energy was transformed into the elastic potential energy of the vascular grafts, buffering and sharing the tensile stress of the blood flow on the aortic root and the AS wall and protecting the STJ.

Adventitial inversion could also increase the strength of the aortic root to some extent. On the contrary, the aortic root was not subjected to special treatment in group B, and there was no clear pressure transition area, with relative solidification of the anastomotic stoma making the upstream a local pressure release area, and the resulting changes in blood flow were highly likely to affect the structure of the aortic root upstream of the anastomotic stoma (16, 17).

Due to the progressive development of reoperation-related events, the long-term implementation of secondary treatment of the aortic root is crucial to effectively reduce any fatal damage caused by reoperation. As such, it has become the long-term focus of clinicians to explore how to ensure the sustainable and stable effect of root reconstruction. As reported in the existing literature, the rate of reoperation due to valvular degeneration was approximately 10–20% when the aortic valve junction suspension technique was used for the reduction of the aortic valve (18, 19). In the present study, there were no differences in aortic regurgitation between the two groups during the postoperative hospitalization period; however, the progression rate of aortic regurgitation in group B during the follow-up period was 34% (17/50), while that in group A was 12.1% (4/33), indicating that adventitial inversion has a better valve maintenance effect than reduction of valve junction suspension.

Comparison of reoperation-related events among the two groups that emerged during the follow-up period indicated that the closure effect of the false lumen of the aortic root in group A was superior to that of the traditional group and the incidence of anastomotic leakage was significantly reduced, while there were no significant differences in other reoperation-related events.

The survival curve obtained in the current study indicated that there were no differences in the short-term survival rate between the two groups of patients. However, the difference in survival rate between the two groups may not have been underestimated due to the short follow-up period. However, comparison of the incident time curve of the first reoperation-related events between the two groups revealed significant differences in terms of effects before and after 12 months, suggesting that the extended adventitial inversion suture and graft eversion anastomosis technique could produce a satisfactory long-term suturing effect, better than that achieved with the aorta wrapping and right atrial shunt technique in terms of aortic root reconstruction, meaning that the reoperation rate of patients in the former group may be lower and their long-term prognosis may be better.

Given that adventitial inversion relies heavily on the adventitia tissue to complete the reinforcement operation, some of the practical limitations of this technique are corresponding revealed, i.e., a too-high requirement for the adventitial integrity. For patients with an oversized false lumen or severe expansion of the aortic root, the supporting effect is often poor due to thin adventitia tissue, and the effect of eliminating the false lumen may not be ideal, which means that other techniques are often required to provide auxiliary support (20).

In conclusion, the use of the extended adventitial inversion suture and graft eversion anastomosis technique in the treatment of AAAD allows for the use of large-area suture surgery and significantly shortens the operation time compared to the method involving a right atrial shunt. Application of an adventitial inversion with graft eversion anastomosis can prevent STJ dilation and is a safe and reliable technique for the treatment of the aortic root, one that can deliver satisfactory short-term postoperative results. Nevertheless, further evaluation is required using a larger sample size and an extended follow-up time period.

## Data availability statement

The original contributions presented in this study are included in the article/supplementary material, further inquiries can be directed to the corresponding author.

## Ethics statement

The studies involving human participants were reviewed and approved by the Ethics Committee of Dalian Municipal Central Hospital. The patients/participants provided their written informed consent to participate in this study.

## Author contributions

FG and XH: conception and design of the research. ZS: acquisition of data, analysis and interpretation of the data, and statistical analysis. XH: obtaining financing. FG and ZS: writing of the manuscript. XH, FG, YG, XZ, LS, WW, and WL: critical revision of the manuscript for intellectual content. All authors read and approved the final draft.
